# Beyond Food Neophobia: Factors Associated with Willingness to Consume Cultured Meat and Edible Insects

**DOI:** 10.3390/foods15213772

**Published:** 2026-10-22

**Authors:** Ayse Derya Bayazit, Mehmet Arif Icer, Dilek Sivri, Ozge Yesildemir, Makbule Gezmen-Karadag

**Affiliations:** 1 Department of Nutrition and Dietetics, Faculty of Health Sciences, Gazi University, 06560 Ankara, Türkiye; 2 Department of Nutrition and Dietetics, Faculty of Health Sciences, Amasya University, 05100 Amasya, Türkiye; 3 Department of Nutrition and Dietetics, Faculty of Health Sciences, Anadolu University, 26470 Eskişehir, Türkiye; 4 Department of Nutrition and Dietetics, Faculty of Health Sciences, Bursa Uludag University, 16059 Bursa, Türkiye

**Keywords:** cultured meat, edible insects, consumer acceptance, willingness to consume, food choice behavior, food neophobia, alternative proteins, awareness, prior experience

## Abstract

This study examined stated willingness to consume cultured meat and edible insects and the associations of unwillingness with food neophobia, sociodemographic characteristics, dietary habits, awareness, and prior consumption experience. This cross-sectional study included 2520 adults aged 18–35 years recruited from urban areas of six cities in Türkiye. Data were collected through face-to-face interviews, in which participants reported their willingness to consume each product. Food neophobia was assessed using the validated Turkish Food Neophobia Scale, and dietary intake using a 24 h dietary recall. Separate binary logistic regression analyses were performed. Stated willingness to consume edible insects and cultured meat was 9.4% and 15.6%, respectively. Disgust (73.2%) and perceived unhealthiness (28.7%) were the main reasons for unwillingness, respectively. Food neophobia was not independently associated with unwillingness to consume cultured meat (adjusted OR = 0.989, 95% CI: 0.977–1.002; *p* > 0.05). For edible insects, food neophobia was significant in the crude model (OR = 1.017, 95% CI: 1.002–1.032; *p* < 0.05), but not after adjustment (adjusted OR = 1.015, 95% CI: 0.998–1.031; *p* > 0.05). Greater general awareness of alternative foods and previous consumption experience of alternative foods were independently associated with lower unwillingness to consume both products (*p* < 0.05). Willingness to consume cultured meat and edible insects appears more closely related to general awareness of alternative foods and previous consumption experience than to general food neophobia. These findings reflect stated willingness rather than observed purchasing or consumption.

## 1. Introduction

Population growth, uneven availability of foods needed for health, and increasing pressure on land, freshwater, and other resources are intensifying interest in affordable, ethical, and sustainable food sources [1,2]. Conventional animal-derived proteins provide high-quality protein and desirable sensory attributes [1,3], but their production can entail substantial land and water use, greenhouse gas emissions, pollution, biodiversity loss, and food-safety challenges [1]. These concerns have increased interest in more sustainable protein-production systems [1].

In this context, alternative protein sources, such as plant-based proteins, single-cell proteins, cultured meat, edible insects, and algae have the potential to contribute to sustainability by reducing greenhouse gas emissions, improving resource efficiency, and mitigating the impacts of climate change [1]. Although alternative food sources have the potential to contribute substantially to sustainable food systems, limitations in the feasibility of industrial-scale production, consumer reluctance, and low public awareness may hinder their widespread adoption [1].

Cultured meat is produced by proliferating animal-derived starter cells under controlled conditions [4]. Its nutritional composition may differ from that of conventional meat and may be modified through culture conditions and product formulation [4]. Prospective life-cycle assessments have suggested that cultured meat could reduce land use and some environmental impacts relative to conventional livestock production; however, the magnitude of these effects depends strongly on production assumptions, scale, and energy sources [5]. Edible insects are also considered an alternative protein source because they provide protein and essential micronutrients while potentially requiring less land and water and generating lower greenhouse gas emissions than conventional livestock systems [1,6]. To increase consumer acceptance, insect-based products can be processed into powders or protein isolates that can be incorporated into foods in ways that minimize their impact on sensory characteristics [6].

Insect food production encompasses wild collection, semi-cultivation, and farming; an FAO report provides examples from Asia, Africa, and Latin America [7]. Culinary examples include crickets, grasshoppers, caterpillars, and beetle larvae. House crickets (Acheta domesticus) and yellow mealworms (Tenebrio molitor) are examples relevant to insect rearing and food preparation [7]. FAO distinguishes insect production for direct human consumption, processed food ingredients, and animal feed [8]; feed production should therefore not be equated with the supply of insects for human diets. Whole insects and processed ingredients represent different food presentations, making appearance and expected sensory properties relevant to consumer evaluation [6,9].

Food neophobia concerns reluctance to consume unfamiliar foods, whereas disgust concerns an aversive response that may also occur toward foods whose identity is known. Sogari et al. examined food neophobia, sensory expectations, previous consumption, and insect-eating intention and behavior in 88 Italian participants [10]. La Barbera et al. found that neophobia and disgust made independent contributions to insect-eating intentions, with a stronger explanatory contribution from disgust [11]. Florença et al. synthesized culturally diverse motivations for insect consumption [9], while Szlachciuk and Żakowska-Biemans examined perceptions, beliefs, willingness to eat insects, and neophobia among Polish adults [12]. An updated review also addresses insect-food acceptance research published during 2021–2024 [13]. These studies establish that neophobia, experience, and product perceptions are existing research themes rather than novel variables introduced here. Product terminology has also been investigated in cultured-meat preference research [14], while situational context has been examined in the acceptance of novel and unfamiliar foods [15].

In parallel with technological advances in alternative food production, consumer acceptance of cultured meat and edible insects, willingness to consume these products, and willingness to pay for them have been investigated in various studies [9,14,15]. Consumer food choices regarding emerging products may reflect an interaction of psychological responses, health and safety perceptions, cultural and social influences, information, familiarity, prior experience, and existing dietary practices [13,16,17,18]. Attitudes such as food neophobia, disgust, and distrust have been reported to reduce willingness to consume alternative food sources [16]. Food neophobia has also been associated with diet quality, energy and nutrient intakes, and food-group consumption [16,19,20,21,22]. However, it remains unclear whether general food neophobia independently contributes to the rejection of specific alternative proteins after informational, experiential, sociodemographic, and dietary factors are considered simultaneously, and whether the pattern differs between cultured meat and edible insects. Therefore, this study aimed to characterize stated willingness to consume cultured meat and edible insects among young adults and to examine the associations of food neophobia, sociodemographic characteristics, dietary habits, awareness of alternative foods, and prior consumption experience with unwillingness to consume these products.

The conceptual framework considered food neophobia, general awareness and previous consumption of alternative foods, sociodemographic characteristics, and dietary intake as potential correlates of unwillingness to consume cultured meat and edible insects, modeled separately for each product, among young adults in six cities in Türkiye. The framework also considered differences in the stated reasons for unwillingness to consume cultured meat and edible insects, which were examined descriptively.

## 2. Materials and Methods

### 2.1. Study Design and Participants

This descriptive cross-sectional study included young adults aged 18–35 years recruited from urban areas of Ankara, İstanbul, Antalya, Eskişehir, Konya, and Kayseri, Türkiye. The age range was selected to focus on an adult population while limiting age-related heterogeneity in food neophobia and willingness to consume alternative foods. Recruitment began on university campuses using convenience sampling and continued through snowball sampling, whereby participants referred other eligible individuals. Both university students and non-students were included. Data were collected through face-to-face interviews between 15 March 2025, and 30 March 2026. Eligibility required being aged 18–35 years, residing in Türkiye during the study period, and voluntarily agreeing to participate with written informed consent. Questionnaires with incomplete core sections, such as the Food Neophobia Scale, alternative-food questions, or dietary recall, were excluded. The final analytical sample comprised 2520 participants.

### 2.2. Data Collection and Questionnaire

Data were collected using a structured questionnaire administered through face-to-face interviews. The questionnaire included items assessing sociodemographic characteristics, self-reported anthropometric data, awareness and previous experience of alternative foods, food neophobia, and dietary intake. The sociodemographic variables included in the present analysis were age, sex, and educational level. Participants self-reported their body weight and height, from which body mass index (BMI) was calculated as weight in kilograms divided by height in meters squared (kg/m^2^). Information on participants’ awareness of alternative foods and previous consumption of alternative foods was also collected. Food neophobia and dietary intake were assessed using specific instruments, as described in detail below. Interviews, including the questionnaire and dietary recall, took approximately 25–30 min. Participation was voluntary, and no financial or non-financial incentives were provided. The questionnaire sections used in this article and their response options are provided in Supplementary Material S1.

### 2.3. Assessment of Awareness, Experience, and Acceptance of Alternative Foods

Participants’ awareness, previous consumption experience, and willingness to consume alternative foods were assessed using structured questionnaire items. Awareness and previous consumption were evaluated in relation to alternative foods in general, with examples including artificial meat, plant-based milk, edible insects, edible flowers, aquatic plants, and microalgae. Participants were also asked about their consumption patterns and reasons for consuming each of these foods, as well as their specific reasons for unwillingness to consume cultured meat and edible insects. The questionnaire used the Turkish term “yapay et” (artificial meat). To ensure conceptual understanding, interviewers explained to all participants that the term referred to meat produced from animal cells. Interviewers used similar explanations in their own words rather than a standardized script.

Willingness to consume was assessed separately for cultured meat and edible insects using dichotomous (yes/no) questions. Participants were asked whether they would be willing to consume cultured meat if it became available in Türkiye and whether they would be willing to try edible insects. Participants who indicated unwillingness were subsequently asked to select one reason for their response for each product. General awareness of and previous experience with alternative foods were included as explanatory variables in the separate regression models examining unwillingness to consume cultured meat and edible insects.

The questionnaire also included two items assessing anticipated greater inclination to consume under a hypothetical health-information scenario: “If you had information about the health effects of artificial meat, would you be more inclined to consume these products?” and “If you had information about the health effects of edible insects, would you be more inclined to consume these products?” No health-related information was provided to participants before these questions were asked. Therefore, the responses were interpreted descriptively as self-reported anticipated greater inclination to consume under a hypothetical condition of being informed and were not considered measures of post-information willingness to consume or evidence of an effect of information provision. The relevant questionnaire items and response options are presented in English in Supplementary Material S1.

### 2.4. Food Neophobia Assessment

Food neophobia was assessed using the validated Turkish version of the Food Neophobia Scale (FNS) [23]. The scale contains 10 items covering willingness to try unfamiliar foods, trust in new foods, responses to unknown ingredients, and interest in foods from other cultures. Responses range from 1 (strongly disagree) to 7 (strongly agree). Items 1, 4, 6, 9, and 10 are worded toward openness to unfamiliar foods and are reverse-scored (reversed score = 8 − response). Scores are summed to give a total of 10–70; higher scores indicate greater food neophobia. Following previously used research categories [24,25], scores of 10–24, 25–39, and 40–70 were designated low, moderate, and high food neophobia, respectively. These are literature-based analytical categories, not clinical diagnostic thresholds. Categories were used for descriptive and dietary group comparisons, whereas the continuous total score was used in logistic regression. Item wording and response options are provided in Supplementary Material S1.

### 2.5. Dietary Intake Assessment

Dietary intake was assessed using a 24 h dietary recall. Participants were asked to report all foods and beverages consumed during the preceding day according to eating occasions, including breakfast, morning snack, lunch, afternoon snack, dinner, and night-time snack. Dietary intake data obtained from the 24 h dietary recalls were analyzed using the full version of the Nutrition Information System (BeBiS), version 7.2 (EBISpro for Windows, Stuttgart, Germany; Turkish version BeBiS) to calculate daily energy and nutrient intakes. Food items were subsequently aggregated into predefined food groups, and food-group intakes were expressed as grams per day (g/day). The food groups evaluated in the present analysis included whole-grain bread, white bread, cereals, milk and yogurt, cheese, eggs, red meat, poultry, fish, legumes, nuts and seeds, fruits, vegetables, solid fats, liquid oils, potatoes, sugary foods, and processed meat. The photographic food portion catalogue by Rakıcıoğlu et al. was used to assist portion-size estimation [26]. The resulting values describe reported intake on the preceding day and were not treated as estimates of individual habitual intake.

### 2.6. Statistical Analysis

The IBM SPSS Statistics 25.0 software package (IBM Corp., Armonk, NY, USA) was used for data analysis. The normality of continuous variables was evaluated using the Kolmogorov–Smirnov test, and dietary intake variables were found to be non-normally distributed. Therefore, the nonparametric Kruskal–Wallis test was used to compare dietary intakes across the low-, moderate-, and high-food-neophobia groups, followed by Bonferroni-adjusted pairwise post hoc comparisons when significant differences were detected. Dietary intake variables are presented as medians (IQR), selected descriptive characteristics as mean ± SD, and categorical variables as *n* (%).

Separate binary logistic regression analyses were performed to identify factors associated with unwillingness to consume cultured meat and unwillingness to try edible insects. For each regression, unwillingness was coded as 1 and willingness as 0. Crude and multivariable models were used to estimate odds ratios (ORs) and adjusted odds ratios (aORs), respectively, with 95% confidence intervals (CIs). The multivariable models included FNS score, age, sex, BMI, educational level, awareness of alternative foods, previous consumption of alternative foods, and intakes of red meat, poultry, potatoes, sugary foods, and processed meat. Covariates were selected based on their conceptual relevance and the preceding analyses. FNS score was the primary variable of interest, while age, sex, BMI, and educational level were potential sociodemographic and anthropometric confounders. Awareness and prior consumption of alternative foods were included to account for prior knowledge and experience. Among the dietary variables assessed, red meat, poultry, potatoes, sugary foods, and processed meat were included because their intakes differed significantly across food-neophobia groups in the preceding Kruskal–Wallis analyses. For ease of interpretation, these dietary intake variables were rescaled to 10 g/day increments in the logistic regression analyses, and the corresponding ORs represent the change in odds per 10 g/day increase in intake. Multicollinearity among the predictors was assessed using tolerance and variance inflation factor (VIF) values. Tolerance values ranged from 0.864 to 0.991, and VIF values ranged from 1.009 to 1.158, indicating no evidence of problematic multicollinearity. Model goodness-of-fit was evaluated using the Hosmer–Lemeshow test, which indicated no statistically significant lack of fit for either the cultured meat model (χ^2^ = 6.803, *p* > 0.05) or the edible insect model (χ^2^ = 3.553, *p* > 0.05). Female sex, primary school/high school education, no awareness of alternative foods, and no previous consumption of alternative foods were used as reference categories. A *p* value < 0.05 was considered statistically significant.

### 2.7. Ethical Considerations

Ethical approval for the study was obtained from the Gazi University Rectorate Ethics Commission on 11 March 2025 (Decision No. 04; Research Code No. 2025-399). The study was conducted in accordance with the ethical principles of the Declaration of Helsinki. All participants were informed about the purpose and procedures of the study, and written informed consent was obtained prior to participation. Participation was voluntary, and participant confidentiality was maintained throughout the study. Study data were recorded and stored using identification codes rather than personally identifiable information.

## 3. Results

The sociodemographic, health, and dietary characteristics of the participants are summarized in Table 1. A total of 2520 participants were included in the study, of whom 53.6% were female and 46.4% were male. The mean age was 24.8 ± 4.9 years, and the mean BMI was 23.8 ± 3.6 kg/m^2^. Using the BMI categories applied in the study, most participants had normal weight (59.6%), while 30.5% were classified as overweight. Most participants were single (75.0%), had a university education (77.0%), and were employed (59.8%). Nearly half had a monthly income between 17,000 and 50,000 TRY (45.9%), and 15.2% of participants with available data had a history of residence abroad. Most had no chronic disease (91.2%) or current medication use (93.9%), whereas dietary supplement use was relatively uncommon (7.7%). Nearly one-third were current smokers (29.8%). Regarding dietary habits, almost all participants (98.1%) followed a traditional dietary pattern. Consumption of two (49.2%) or three (48.2%) main meals per day was most common, while 21.4% did not consume snacks. The mean daily water intake was 1781.7 ± 747.1 mL, and only 5.4% were currently following a diet.

The awareness, experience, perceptions, and willingness to consume alternative food sources are presented in Table 2. Overall, 52.3% of participants were aware of alternative food sources, whereas only 15.3% had previously consumed at least one such product. Among those with prior experience, plant-based milk was the most frequently consumed alternative food source (76.4%), and nutritional value was the main reason for consumption (70.4%). Most participants who had consumed an alternative food source reported accepting its taste (76.2%). Fourteen participants (3.6% of those who reported previous consumption of alternative foods) self-reported having consumed cultured meat.

Willingness to consume cultured meat (15.6%) and edible insects (9.4%) was generally low. The most common reason for unwillingness to consume cultured meat was the perception that it is unhealthy (28.7%), whereas disgust was the predominant reason for rejecting edible insects (73.2%). Only 8.4% and 8.0% of participants perceived cultured meat and edible insects, respectively, to be as safe and healthy as conventional animal foods. When asked whether they would be more inclined to consume the products if informed about their health effects, 30.8% answered yes for cultured meat and 11.2% for edible insects. Willingness was assessed hypothetically, without providing participants with additional information about the products; therefore, the responses do not reflect willingness after exposure to information (e.g., on safety, nutritional value, or sustainability). Only 12.7% of participants perceived alternative food sources as having nutritional content similar to that of conventional foods, and 8.3% were willing to replace conventional foods with alternative sources. More than half of the participants (54.5%) believed that the consumption of alternative food sources would increase in the future, whereas only 14.6% considered these foods beneficial for health. Regarding willingness to pay for healthier personalized foods, 52.2% of participants were willing to pay up to 50% more.

**Figure 1:**
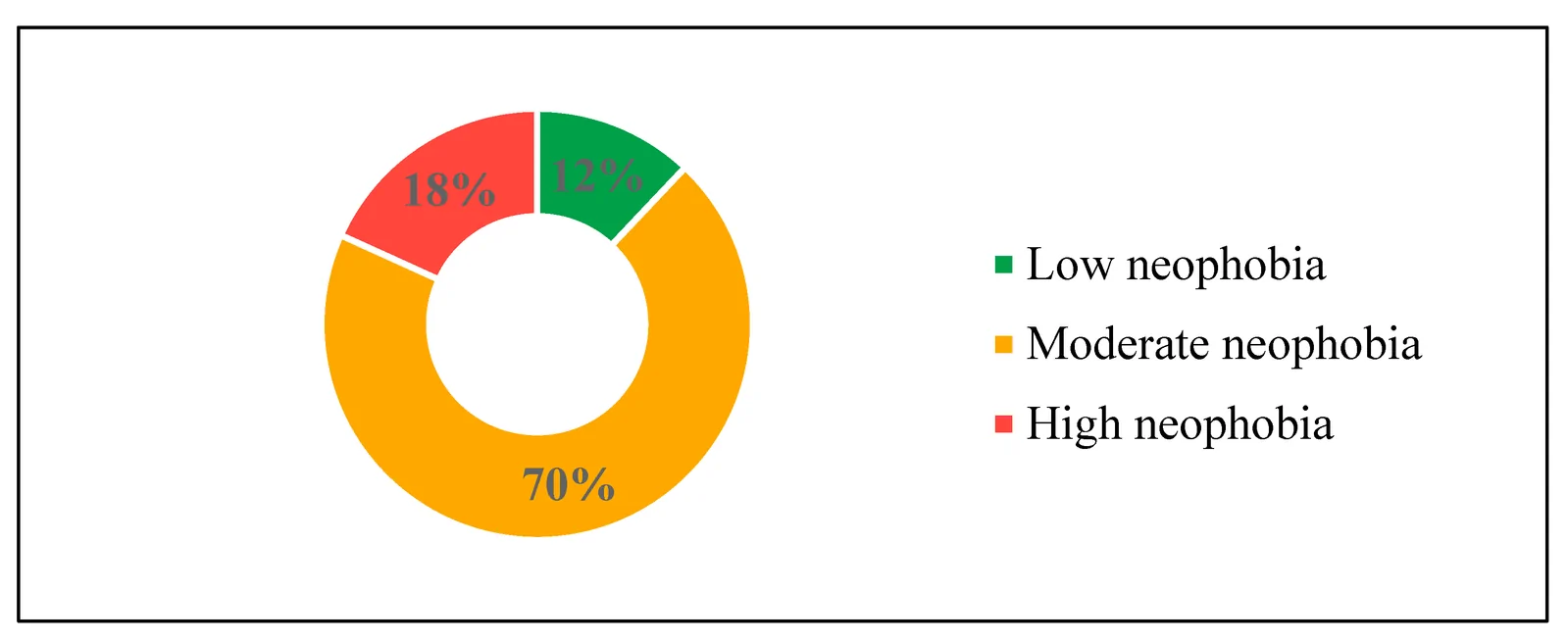
Distribution of food neophobia levels.

The mean Food Neophobia Scale (FNS) score was 37.62 ± 9.33 (range: 10.0–70.0). Based on the FNS classification, participants were categorized into three food neophobia groups: low (12.0%, *n* = 303), moderate (69.8%, *n* = 1758), and high (18.2%, *n* = 459) (Figure 1).

Table 3 presents the comparison of daily energy and nutrient intake according to food neophobia level. No statistically significant differences were observed among the low-, moderate-, and high-food-neophobia groups for the nutrient and diet-quality variables presented (all *p* > 0.05).

**Figure 2:**
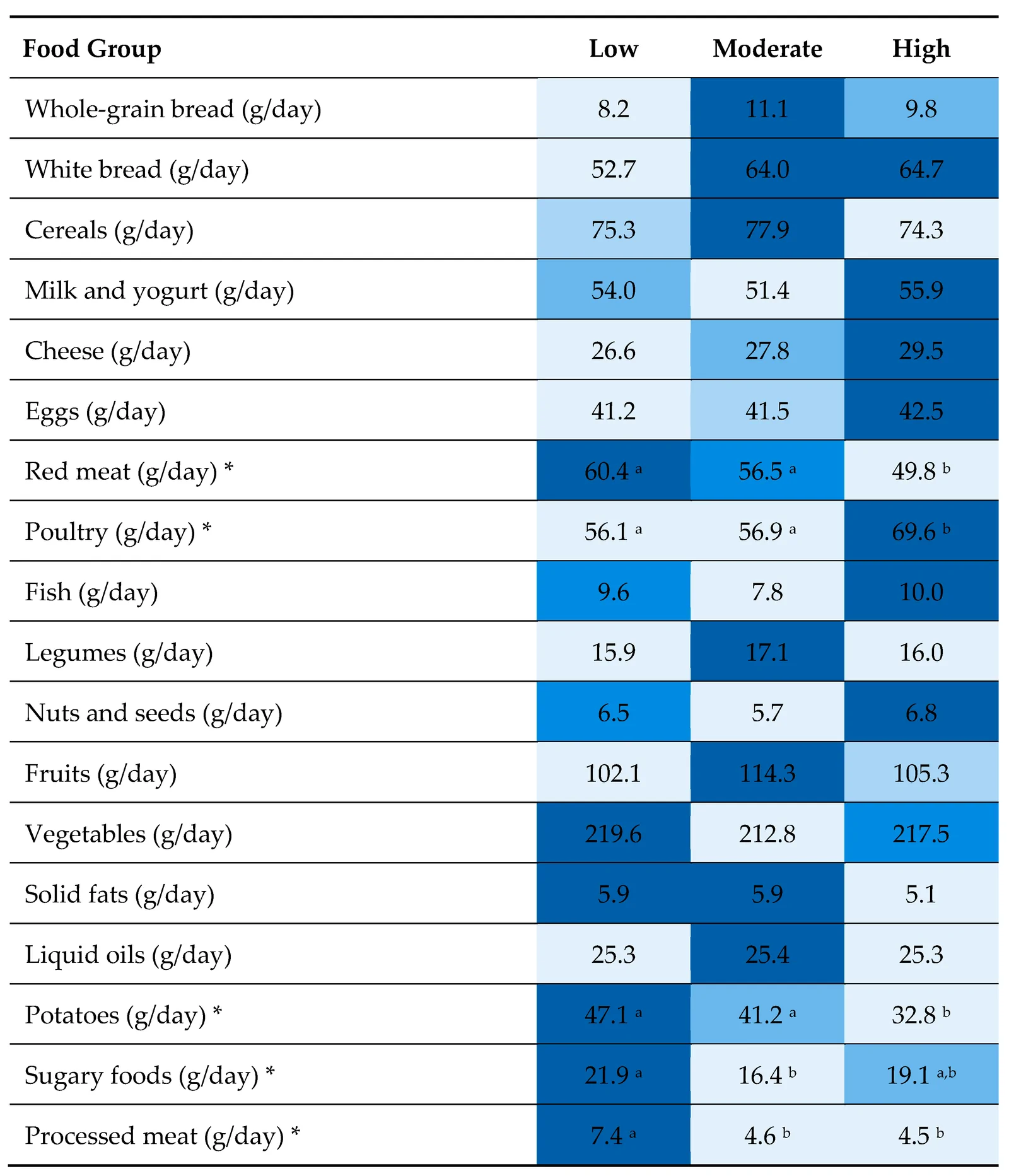
Mean daily intake of all food groups (g/day) across food neophobia levels. * Statistically significant difference across neophobia groups (*p* < 0.05, Kruskal–Wallis test). Mean values are presented to facilitate interpretation of average intake levels. Superscript letters (a, b) indicate pairwise post hoc comparisons; groups sharing the same letter are not significantly different. Color shading reflects relative intake within each food group (row); darker shading indicates higher intake.

Mean daily intake of food groups across food neophobia levels is presented in Figure 2. Significant differences were observed in the consumption of red meat, poultry, potatoes, sugary foods, and processed meat (all *p* < 0.05), whereas intake of the remaining food groups did not differ significantly between food-neophobia groups (all *p* > 0.05). Red meat consumption was highest in the low-neophobia group and lowest in the high-neophobia group (60.4 ± 66.6 vs. 49.8 ± 62.5 g/day, *p* < 0.05). In contrast, poultry intake increased with increasing food neophobia, with the highest consumption observed in the high-neophobia group (69.6 ± 98.0 g/day) compared with the low- and moderate-neophobia groups (*p* < 0.05). Potato intake showed the opposite pattern, declining progressively from the low- to the high-neophobia group (47.1 ± 72.0 vs. 32.8 ± 60.4 g/day, *p* < 0.05). Sugary food intake did not follow a linear trend, with the moderate-neophobia group showing the lowest intake. The omnibus Kruskal–Wallis test was significant (*p* < 0.05), but the high-neophobia group did not differ significantly from either the low- or moderate-neophobia group in pairwise comparisons. Processed meat consumption decreased with increasing food neophobia, from 7.4 ± 28.2 g/day in the low-neophobia group to 4.5 ± 27.5 g/day in the high-neophobia group (*p* < 0.001).

Table 4 presents the crude and adjusted logistic regression models examining factors associated with unwillingness to consume cultured meat. Male participants had lower odds of unwillingness than female participants in both the crude (OR = 0.783, *p* < 0.05) and adjusted models (adjusted OR = 0.668, 95% CI: 0.532–0.853, *p* < 0.05). Likewise, participants with a university-level education were less likely to report unwillingness than those with primary school/high school education (adjusted OR = 0.670, 95% CI: 0.476–0.943, *p* < 0.05). Awareness of alternative food sources (adjusted OR = 0.365, 95% CI: 0.278–0.480, *p* < 0.001) and prior consumption of alternative foods (adjusted OR = 0.288, 95% CI: 0.220–0.375, *p* < 0.001) showed the strongest adjusted associations, indicating substantially lower odds of unwillingness among participants who were aware of or had previously consumed alternative food sources. Among food-group intake variables from the 24 h recall, higher potato (adjusted OR = 1.027 per 10 g/day, 95% CI: 1.006–1.048, *p* < 0.05) and sugary food consumption (adjusted OR = 1.045 per 10 g/day, 95% CI: 1.001–1.090, *p* < 0.05) were linked to modestly higher odds of unwillingness, although the effect sizes were small.

Table 5 presents the crude and adjusted logistic regression models examining factors associated with unwillingness to try edible insects. Although a higher FNS score was associated with greater odds of unwillingness in the crude model (OR = 1.017, 95% CI: 1.002–1.032, *p* < 0.05), this association was no longer significant after adjustment (adjusted OR = 1.015, 95% CI: 0.998–1.031, *p* > 0.05). Male participants had lower odds of unwillingness than female participants (adjusted OR = 0.597, 95% CI: 0.446–0.801, *p* < 0.001). Awareness of alternative food sources (adjusted OR = 0.546, 95% CI: 0.402–0.742, *p* < 0.001) and prior consumption of alternative foods (adjusted OR = 0.683, 95% CI: 0.483–0.967, *p* < 0.05) showed the strongest adjusted associations, indicating substantially lower odds of unwillingness among participants who were aware of or had previously consumed alternative food sources. Among food-group intake variables from the 24 h recall, only higher sugary food consumption was linked to lower odds of unwillingness (adjusted OR = 0.960 per 10 g/day, 95% CI: 0.925–0.996, *p* < 0.05). University-level education was not independently associated with unwillingness after adjustment.

## 4. Discussion

In this cross-sectional study conducted among young adults, the consumption of plant-based alternatives was higher than that of cultured meat and edible insects. The most common reason for unwillingness to consume cultured meat was the perception that these products were unhealthy, whereas disgust was the predominant reason for rejecting edible insects. In multivariable analyses, food neophobia was not significantly associated with unwillingness to consume cultured meat or edible insects, whereas awareness of alternative foods and prior consumption experience were independently associated with lower unwillingness to consume both products.

Consumer acceptance of animal-derived alternative protein sources has been reported to be lower than that of plant-based alternatives [27,28]. A systematic review reported that acceptance of alternative proteins was lower than that of conventional meat and that, among alternative protein sources, edible insects had the lowest acceptance, followed by cultured meat, whereas plant-based proteins had the highest acceptance [27]. In our study, among the 386 participants who reported prior consumption of alternative foods, plant-based milk was the most frequently selected product (76.4%, *n* = 295). In addition, 52.3% were aware of alternative food sources, whereas only 15.3% had tried these foods at least once. In another study in which young adults constituted the majority of the sample, 60.0% of participants were aware of alternative proteins; however, only 32.0% had tried them at least once, and 15.0% consumed them regularly [29]. These findings suggest that awareness of alternative food sources may be more widespread than actual consumption experience.

In our study, 9.4% of participants were willing to consume edible insects. A study conducted among young adults in Japan reported that 15.9% of participants had previously consumed edible insects and 22.0% were willing to consume insects in the future [30]. In the present study, the most prominent reason for rejecting edible insects was disgust (73.2%), which is consistent with previous research [31,32]. However, curiosity about the taste of edible insects has also been reported to encourage consumers to try these products [30]. It has also been shown that consumers prefer products in which edible insects are not visible and that products in which insects are visually identifiable are associated with more negative sensory experiences [32].

The prominence of disgust for insects can be interpreted alongside research separating general neophobia from product-related aversion [11]. Neophobia may reflect hesitation toward novelty, while learned associations may contribute to disgust even when the product is recognized. Cultural food categories, memories of previous experiences, and expectations about appearance, texture, flavor, or odor offer possible explanations for acceptance or rejection [7,9,10,11,12]. However, this study did not measure memories, implicit associations, or sensory responses through tasting. Its single-choice rejection questions identify a selected reason, not a validated measure of emotional intensity or all concurrent barriers. These mechanisms should therefore be treated as literature-informed interpretations requiring direct investigation.

In our study, 15.6% of participants were willing to consume cultured meat. Similarly, a previous study reported that 20.2% of participants were willing to consume cultured meat [29]. In contrast, higher levels of willingness have been reported in other populations, with 57–66% of participants willing to try cultured meat and 43–57% willing to consume it regularly [33,34,35]. These differences suggest that acceptance of cultured meat may vary across countries, cultural contexts, religious beliefs, and dietary habits. In our study, the most common reason for unwillingness to consume cultured meat was the perception that the product was unhealthy (28.7%); moreover, only 8.4% of participants considered cultured meat and 8.0% considered edible insects to be as safe and healthy as conventional animal-based foods. Previous studies have identified concerns regarding naturalness, health and safety, expected sensory characteristics, price, trust, and familiarity as barriers to the acceptance of cultured meat [34,36,37]. Similarly, uncertainties regarding the health and safety characteristics of cultured meat may influence consumer perceptions [4,5,38,39], while concerns about heavy metals, toxins, and pesticide residues in edible insects may limit consumer acceptance [32,40]. These findings suggest that the low willingness to consume both alternative protein sources may, at least in part, be associated with low confidence in their health and safety characteristics.

Only 12.7% of participants considered the nutritional content of alternative food sources to be similar to that of conventional foods, and 8.3% reported willingness to replace conventional foods with alternative sources. Nevertheless, 54.5% expected alternative-food consumption to increase in the future. In addition, 52.2% reported stated willingness to pay up to 50% more for healthier personalized foods. Because this willingness-to-pay item concerned healthier personalized foods generally rather than cultured meat or edible insects specifically, it should be interpreted as a broader, hypothetical health-oriented preference and not as willingness to pay for the products studied. Previous research likewise indicates that consumer interest in alternative proteins can coexist with limited knowledge and uncertainty, while confidence in their health, sustainability, and environmental attributes may support willingness to consume them [27,29]. Accurate, understandable, and alternative food-specific information may therefore help consumers evaluate unfamiliar foods, although information alone may be insufficient when rejection is driven by disgust or other affective responses.

Food neophobia, together with distrust and disgust, is considered one of the important attitudinal determinants of willingness to consume and pay for alternative food sources [27]. In our study, the mean FNS score was 37.62 ± 9.33, which is comparable to the moderate levels of food neophobia reported in previous studies [41,42]. When evaluated according to food neophobia levels, the higher consumption of red meat in the low-neophobia group was consistent with a previous study conducted in Türkiye [43], whereas the higher poultry consumption observed in the high-neophobia group was not consistent with the findings of that study [43]. Although processed meat consumption decreased with increasing neophobia in our study, the previous study conducted in Türkiye reported no significant association between processed meat consumption and neophobia groups [43].

High food neophobia has been associated with lower dietary diversity and diet quality [19], particularly with lower fruit and vegetable consumption [20,21]. Neophobic individuals may also be less likely to prefer a variety of foods, including meat, fish, fruits, and vegetables [44]. Some studies have linked higher food neophobia with greater intake of energy-dense or sugary foods [20,45,46], whereas another reported no association [21]. In our study, however, consumption of vegetables, fruits, fish, and whole grains did not differ significantly across food-neophobia levels. Furthermore, energy, macronutrient and micronutrient intakes, and diet-quality indicators were not significantly associated with food neophobia. Because these findings were obtained using dietary intake assessed with a single 24 h dietary recall method, they may not fully reflect habitual food intake, dietary variety, or overall diet quality, and this may have affected the results.

Although individuals with high food neophobia have been reported to be less willing to try alternative food sources [16,22], food neophobia was not independently associated with unwillingness to consume cultured meat in the present study. This finding is consistent with previous studies suggesting that food neophobia may have limited predictive value for cultured meat acceptance and that emotional and perceptual evaluations specific to cultured meat may more strongly explain consumption intentions [47]. In contrast, a multinational study involving 3091 participants showed that food neophobia negatively affected willingness to try and purchase cultured meat and to pay a higher price for it [16]. In addition, food technology neophobia has been reported to be associated with intentions to try and regularly purchase cultured meat [48]. These differing findings suggest that the role of general food neophobia in cultured meat acceptance may vary across populations, cultural contexts, and the consumer behaviors being assessed. Nevertheless, the present findings indicate that product and production technology-specific perceptions should be considered alongside general food neophobia when evaluating willingness to consume cultured meat.

When evaluated in terms of demographic characteristics, men in our study had lower odds of unwillingness to consume cultured meat than women. Associations between sex and food neophobia or cultured-meat acceptance have varied across populations and study designs [46,49,50,51]. Regarding education level, individuals with a university/postgraduate education had 33.0% lower odds of being unwilling to consume cultured meat than those with primary/high school education (OR = 0.670; 95% CI: 0.476–0.943; *p* = 0.022). A previous study also reported that individuals with lower education levels were less willing to try cultured meat [34]. Studies reporting no association between age and willingness to consume cultured meat [34] are consistent with our findings, whereas other studies have shown that willingness decreases with increasing age [28,35]. These differences suggest that the role of demographic characteristics in willingness to consume cultured meat may vary across populations.

A meta-analysis has shown that perceptions specific to cultured meat may be more strongly associated with willingness to consume than individual and demographic characteristics [17]. In this context, it is also noteworthy that the amounts of red meat and poultry consumed were not associated with unwillingness to consume cultured meat in the present study. Similarly, a study conducted in Germany found no association between the amount of meat consumed and willingness to consume cultured meat [52]. This suggests that willingness to consume cultured meat may be more closely related to cultured meat-specific perceptions, attitudes, and evaluations of the technology than to current levels of meat consumption.

Regarding edible insects, food neophobia was significantly associated with unwillingness to consume them in the crude analysis. However, this association was no longer statistically significant after adjustment for sociodemographic characteristics, awareness, prior consumption experience, and dietary habits. In a study conducted among 601 participants in Australia, higher food neophobia was associated with less prior experience of insect consumption and lower willingness to consume insects in the future [18]. A systematic review evaluating motivations for edible insect consumption also reported that younger age, male sex, higher education, greater knowledge, prior experience, and lower food neophobia were associated with greater willingness to consume edible insects [9]. The difference between the crude and multivariable analysis results in our study suggests that the influence of food neophobia on willingness to consume edible insect should be evaluated in conjunction with awareness, prior experience, and other consumer characteristics.

In our study, men had lower unwillingness to consume edible insects than women. Although higher education level was associated with lower unwillingness in the crude analysis, this association lost statistical significance in the multivariable model. In contrast, awareness of alternative foods and prior consumption of alternative foods remained independently associated with lower unwillingness. These findings are consistent with previous studies showing that awareness and prior knowledge are associated with the acceptance of novel protein sources [13,18]. The finding that education level was no longer significantly associated with unwillingness after adjustment suggests that more direct consumer characteristics, such as awareness and prior consumption experience, may be more relevant to edible insect acceptance.

Finally, an unexpected inverse association was observed between sugary food consumption and unwillingness to consume edible insects, meaning that higher sugary food intake was associated with lower unwillingness. However, the magnitude of this association was small, and evidence regarding the relationship between sugary food consumption and willingness to consume alternative foods remains limited. Given the number of dietary variables tested and the use of a single 24 h recall, this finding may reflect chance or measurement limitations and should be interpreted cautiously. Although direct evidence on this relationship is scarce, previous studies examining dietary intake in relation to food neophobia have reported a positive association between food neophobia and energy intake from sugary foods [20,45,46], whereas another study found no significant association [21]. Therefore, this finding requires confirmation in future studies. Although statistically significant, the associations observed for potato and sugary food intake in the cultured meat model and sugary food intake in the edible insect model corresponded to relatively small changes in odds per 10 g/day increase in intake. Their practical or behavioral relevance remains uncertain, particularly because dietary intake was assessed using a single 24 h recall and these exploratory findings cannot be interpreted as reflecting habitual dietary patterns.

### 4.1. Strengths and Limitations

One of the major strengths of this study is the simultaneous assessment of food neophobia, dietary intake, awareness, prior consumption experience, and willingness to consume cultured meat and edible insects in a relatively large sample of 2520 young adults in Türkiye. The collection of data through face-to-face interviews enabled the systematic assessment of multiple dimensions of consumer attitudes using a structured questionnaire. In addition, the use of the validated Turkish version of the Food Neophobia Scale and the detailed assessment of dietary intake in terms of food groups, energy intake, and nutrient intakes are among the methodological strengths of the study. The use of multivariable models to evaluate factors associated with unwillingness to consume cultured meat and edible insects also allowed food neophobia, sociodemographic characteristics, awareness, prior consumption experience, and dietary habits to be considered simultaneously and independent associations to be identified. Furthermore, modeling unwillingness to consume cultured meat and edible insects separately made it possible to compare the factors associated with willingness to consume each product. The contribution of this study is the assessment of both products (cultured meat and edible insects) within the same recruited sample, including their contrasting selected rejection reasons.

However, this study has several limitations. First, its cross-sectional design precludes causal inferences regarding relationships among food neophobia, dietary intake, awareness, prior consumption experience, and attitudes toward alternative foods. Convenience sampling and the inclusion of only individuals aged 18–35 years may limit representativeness for the wider population of Türkiye and generalizability to other age and sociodemographic groups. Face-to-face interviews may also have introduced social desirability bias. Dietary intake was assessed using a single 24 h dietary recall and may not represent usual intake or day-to-day variation, while self-reported weight and height may have introduced measurement error in BMI. Awareness, prior consumption, and willingness were measured with structured self-report questions rather than comprehensive psychometric instruments. Moreover, awareness referred to alternative foods broadly and may not capture product-specific knowledge or experience. Reports of previous cultured-meat or edible-insect consumption were not independently verified; therefore, product identity and the circumstances of consumption remain uncertain. Another limitation is that this study assessed stated willingness to consume cultured meat and edible insects rather than observed purchasing or consumption behavior. Although previous studies often describe such outcomes as “acceptance,” the present findings should be interpreted as reflecting hypothetical willingness only. For participants without previous experience of the products, responses were necessarily based on perceptions, expectations, and hypothetical choice, and may not translate into actual behavior. Willingness was also assessed without providing additional information about the products, which may have influenced responses. In addition, face-to-face data collection may have introduced social desirability bias. Recruitment from six urban settings does not imply national representativeness; the university-based starting points and high proportion of university-educated participants further limit generalization. The hypothetical health questions did not assess an information effect. Unmeasured cultural, religious, economic, emotional, and product-specific factors may contribute to residual confounding. The relatively small willing subgroup for insects (*n* = 238) warrants caution regarding estimate precision, particularly in sparse predictor categories. Separate product-specific regression models do not constitute a formal statistical test of differences in associations between cultured meat and edible insects; any comparison of the patterns observed across the two models should therefore be interpreted descriptively.

### 4.2. Implications for Consumer Food Choice and Future Research

The integration of alternative proteins into sustainable food systems requires consumer-choice strategies that reflect the distinct barriers associated with each product [1]. Some studies show an increase in the desire to consume cultured meat after being given health-related information [53,54], supporting the view that information-based approaches can promote consumer acceptance. Future studies could test whether communication addressing the health, safety, and trust concerns identified in this sample is associated with changes in willingness to consume cultured meat. For edible insects, future studies could test information combined with sensory and product-design approaches, including familiar formats in which insects are not visually identifiable [55]. Future studies should incorporate product- and technology-specific neophobia, disgust, perceived naturalness, labeling, price, trust, and cultural norms; measure actual tasting, purchasing, or repeated consumption where feasible; and examine heterogeneity across sociodemographic groups. More representative recruitment and examination of differences across sociodemographic groups would clarify applicability beyond this sample, while repeated 24 h dietary recalls would strengthen the dietary assessment. Finally, to facilitate the safe and widespread adoption of alternative proteins, further evidence is needed regarding potential allergens, food safety, and appropriate labeling practices, and consumer acceptance should be evaluated across different sociodemographic groups [56].

## 5. Conclusions

Among young adults in Türkiye, stated willingness to consume cultured meat and edible insects was low (15.6% for cultured meat and 9.4% for edible insects), and the main reasons for unwillingness differed between the two products. This study suggests that willingness to consume these products may be more closely related to general awareness of alternative foods and previous consumption experience than to general food neophobia. In multivariable analyses, food neophobia was not independently associated with unwillingness to consume either cultured meat or edible insects, whereas greater general awareness of alternative foods and previous consumption experience were independently associated with lower unwillingness. Perceived unhealthiness was the leading barrier for cultured meat, while disgust predominated for edible insects. The reported reasons for unwillingness provide a basis for future studies testing product-specific approaches to health and safety communication and to disgust-related barriers; the effectiveness of such approaches was not evaluated in this study. Because the study assessed stated willingness rather than observed purchasing or consumption, future experimental and longitudinal studies should investigate whether targeted health and safety information, increased awareness, and familiarization with alternative foods can increase willingness to consume and translate into actual consumption behavior.

## Figures and Tables

**Table 1:** Sociodemographic, health, and dietary characteristics of the participants.

Characteristic	Total (*n* = 2520)
Age (years), mean ± SD	24.8 ± 4.9
Sex, *n* (%)	
Female	1351 (53.6)
Male	1169 (46.4)
Marital status, *n* (%)	
Single	1889 (75.0)
Married	631 (25.0)
Education level, *n* (%)	
Primary school or below	52 (2.0)
High school	441 (17.5)
University	1940 (77.0)
Postgraduate	87 (3.5)
Employment status, *n* (%)	
Employed	1506 (59.8)
Unemployed	1014 (40.2)
Income level, *n* (%)	
0–17,000 TRY	503 (20.0)
17,000–50,000 TRY	1157 (45.9)
50,001–99,999 TRY	569 (22.6)
100,000 TRY and above	291 (11.5)
History of residence abroad, *n* (%)	
Yes	382 (15.2)
No	2130 (84.8)
Duration of residence abroad (days; *n* = 382), mean ± SD	262.9 ± 1165.5
BMI (kg/m^2^), mean ± SD	23.8 ± 3.6
BMI classification, *n* (%)	
Underweight (<18.5 kg/m^2^)	119 (4.7)
Normal (18.5–24.9 kg/m^2^)	1501 (59.6)
Overweight (25.0–29.9 kg/m^2^)	768 (30.5)
Obese (≥30.0 kg/m^2^)	132 (5.2)
Chronic disease status, *n* (%)	
Yes	222 (8.8)
No	2298 (91.2)
Current medication use, *n* (%)	
Yes	153 (6.1)
No	2367 (93.9)
Smoking status, *n* (%)	
Yes	751 (29.8)
No	1769 (70.2)
Current dietary supplement use, *n* (%)	
Yes	193 (7.7)
No	2327 (92.3)
Dietary pattern, *n* (%)	
Traditional	2473 (98.1)
Vegetarian	24 (1.0)
Vegan	4 (0.2)
Flexitarian	19 (0.7)
Number of main meals, *n* (%)	
One	65 (2.6)
Two	1241 (49.2)
Three	1214 (48.2)
Number of snacks, *n* (%)	
None	539 (21.4)
One	907 (36.0)
Two	814 (32.3)
Three	260 (10.3)
Water intake (mL/day), mean ± SD	1781.7 ± 747.1
Currently following a diet, *n* (%)	
Yes	135 (5.4)
No	2385 (94.6)

BMI, body mass index; SD, standard deviation. History of residence abroad was available for 2512 participants; percentages were calculated using available data. Duration is reported among participants with a history of residence abroad (*n* = 382). TRY, Turkish lira. For approximate international comparison, EUR equivalents use the Central Bank of the Republic of Türkiye EUR foreign-exchange buying rate on 30 March 2026 (1 EUR = 51.0236 TRY): 17,000 TRY ≈ 333 EUR; 50,000 TRY ≈ 980 EUR; 100,000 TRY ≈ 1960 EUR. These conversions are illustrative and do not account for exchange-rate or purchasing-power changes during recruitment.

**Table 2:** Awareness, experience, perceptions, and willingness to consume alternative food sources.

Characteristic	Total (*n* = 2520)	
	*n*	%
Awareness of alternative food sources, *n* (%)		
Yes	1317	52.3
No	1203	47.7
Source of information *, *n* (%)		
Digital media (Internet/social media)	994	75.5
Traditional media (television/news)	68	5.2
Education (school/courses/university)	73	5.5
Family and friends	49	3.7
Professional sources	67	5.1
Multiple sources	55	4.2
Other/unclear	11	0.8
Prior consumption of alternative food sources, *n* (%)		
Yes	386	15.3
No	2134	84.7
Type of alternative food source consumed *^,†^, *n* (%)		
Edible insects	11	2.8
Cultured meat	14	3.6
Plant-based milk	295	76.4
Microalgae	10	2.6
Edible flowers	143	37.0
Reasons for consuming alternative food sources *, *n* (%)		
Curiosity	65	16.8
Health benefits	27	7.0
Nutritional value	272	70.4
Vegetarian/vegan diet	6	1.6
Environmental sustainability	8	2.1
Medical reasons	8	2.1
Taste acceptance *, *n* (%)		
Yes	294	76.2
No	92	23.8
Willingness to consume cultured meat, *n* (%)		
Yes	392	15.6
No	2128	84.4
Reason for unwillingness to consume cultured meat *, *n* (%)		
Disgust	476	22.4
Expected unpleasant taste	398	18.6
Religious concerns	190	8.9
Perceived lack of nutritional value	423	19.9
Perceived as unhealthy	610	28.7
Other	31	1.5
Perceived safety and healthiness of cultured meat, *n* (%)		
Yes	211	8.4
No	2309	91.6
Anticipated greater inclination to consume cultured meat if informed about its health effects, *n* (%)		
Yes	775	30.8
No	1745	69.2
Willingness to consume edible insects, *n* (%)		
Yes	238	9.4
No	2282	90.6
Reason for unwillingness to consume edible insects *, *n* (%)		
Disgust	1671	73.2
Expected unpleasant taste	271	11.9
Religious concerns	125	5.5
Perceived lack of nutritional value	71	3.1
Perceived as unhealthy	131	5.7
Other	13	0.6
Perceived safety and healthiness of edible insects, *n* (%)		
Yes	201	8.0
No	2319	92.0
Anticipated greater inclination to consume edible insects if informed about their health effects, *n* (%)		
Yes	283	11.2
No	2237	88.8
Perceived nutritional similarity of alternative and conventional foods, *n* (%)		
Yes	320	12.7
No	2200	87.3
Willingness to replace conventional foods with alternative food sources, *n* (%)		
Yes	208	8.3
No	2312	91.7
Perceived future increase in alternative food consumption, *n* (%)		
Yes	1374	54.5
No	1146	45.5
Perceived health benefits of alternative food sources, *n* (%)		
Yes	368	14.6
No	2152	85.4
Additional amount willing to pay for healthier personalized foods, *n* (%)		
0%	921	36.5
Up to 50%	1316	52.2
50–100%	232	9.2
>100%	51	2.0

* Percentages for items marked with a subgroup denominator are calculated based on the relevant subgroup, not the total sample. † Participants could select more than one type of alternative food source consumed; therefore, percentages may exceed 100%. Reasons for unwillingness were single-response items. Health-related inclination questions were hypothetical; no health information was provided.

**Table 3:** Comparison of daily energy and nutrient intake according to food neophobia level.

Variables	Low (*n* = 303)	Moderate (*n* = 1758)	High (*n* = 459)	*p*
	Median (IQR)	Median (IQR)	Median (IQR)	
Energy and macronutrients				
Energy (kcal/day)	1657.7 (835.6)	1643.9 (809.4)	1592.6 (806.7)	0.514
Protein (g/day)	64.3 (37.9)	65.2 (34.0)	64.3 (34.1)	0.892
Protein (% of energy)	16.0 (6.0)	16.0 (5.0)	16.0 (6.0)	0.065
Fat (g/day)	76.3 (50.6)	72.5 (44.5)	69.5 (47.2)	0.255
Fat (% of energy)	41.0 (11.0)	41.0 (12.0)	41.0 (13.0)	0.351
Carbohydrate (g/day)	166.6 (99.1)	169.8 (96.2)	164.9 (95.4)	0.605
Carbohydrate (% of energy)	42.0 (12.0)	43.0 (13.0)	41.0 (14.0)	0.184
Total dietary fiber (g/day)	16.8 (9.8)	17.7 (11.1)	17.1 (11.3)	0.104
Soluble fiber (g/day)	5.4 (3.3)	5.7 (3.6)	5.4 (3.6)	0.147
Insoluble fiber (g/day)	10.9 (6.2)	11.3 (6.9)	10.4 (7.0)	0.065
Plant protein (g/day)	27.5 (13.6)	28.2 (16.0)	27.4 (16.2)	0.418
Saturated fatty acids (g/day)	23.7 (17.6)	23.1 (16.3)	22.6 (17.3)	0.291
Cholesterol (mg/day)	317.7 (265.1)	300.9 (266.2)	305.8 (296.2)	0.536
Monounsaturated fatty acids (g/day)	26.1 (15.6)	24.7 (15.4)	24.3 (15.6)	0.492
Polyunsaturated fatty acids (g/day)	17.5 (15.9)	16.9 (13.9)	16.5 (13.8)	0.771
Omega-3 fatty acids (g/day)	0.9 (0.9)	0.9 (0.9)	0.9 (0.9)	0.373
Omega-6 fatty acids (g/day)	16.2 (15.0)	15.5 (13.0)	15.2 (12.8)	0.698
Vitamins/Minerals				
Vitamin A (µg/day)	700.4 (956.8)	718.5 (795.7)	652.5 (696.0)	0.167
Vitamin D (µg/day)	1.7 (2.1)	1.5 (1.9)	1.6 (2.2)	0.059
Vitamin E (mg/day)	18.7 (15.0)	18.3 (14.0)	18.1 (14.0)	0.517
Vitamin K (µg/day)	52.6 (90.9)	52.8 (79.4)	55.3 (87.8)	0.245
Vitamin B_1_ (mg/day)	0.8 (0.5)	0.8 (0.5)	0.8 (0.5)	0.287
Vitamin B_2_ (mg/day)	1.2 (0.6)	1.2 (0.7)	1.2 (0.6)	0.796
Niacin (mg/day)	24.8 (19.0)	23.9 (17.6)	25.3 (19.1)	0.245
Pantothenic acid (mg/day)	4.5 (2.4)	4.6 (2.5)	4.7 (2.5)	0.820
Vitamin B_6_ (mg/day)	1.3 (0.8)	1.3 (0.8)	1.3 (0.8)	0.856
Biotin (µg/day)	39.6 (27.6)	39.8 (23.9)	39.9 (24.7)	0.999
Folate (µg/day)	242.6 (175.1)	255.7 (178.1)	248.3 (175.7)	0.455
Vitamin B_12_ (µg/day)	4.3 (5.0)	3.9 (4.6)	3.4 (4.7)	0.060
Vitamin C (mg/day)	98.9 (107.3)	91.9 (94.3)	89.6 (100.0)	0.175
Sodium (mg/day)	1227.6 (936.9)	1221.5 (920.6)	1204.3 (825.2)	0.908
Calcium (mg/day)	518.9 (332.9)	522.6 (340.9)	530.5 (322.5)	0.859
Magnesium (mg/day)	216.5 (120.4)	232.2 (130.7)	223.8 (117.7)	0.544
Phosphorus (mg/day)	962.6 (463.4)	984.6 (487.9)	969.4 (498.9)	0.814
Iron (mg/day)	9.3 (5.9)	9.5 (5.6)	9.3 (5.4)	0.632
Zinc (mg/day)	9.2 (6.1)	9.0 (5.2)	8.7 (5.5)	0.430
Copper (mg/day)	1.2 (0.9)	1.2 (0.8)	1.2 (0.7)	0.658
Selenium (µg/day)	13.5 (22.0)	13.5 (20.0)	13.5 (22.0)	0.886
Diet quality indicators				
Glycemic index	106.0 (99.7)	94.7 (104.4)	106.0 (97.7)	0.072
ORAC	2422.5 (3003.4)	2585.9 (3915.2)	2171.6 (3754.6)	0.230
Dietary antioxidant capacity (mmol/day)	1.4 (1.5)	1.3 (1.5)	1.2 (1.6)	0.221

IQR, interquartile range; ORAC, oxygen radical absorbance capacity. *p* values were obtained using the Kruskal–Wallis test.

**Table 4:** Factors associated with unwillingness to consume cultured meat: crude and adjusted logistic regression models.

Variables	Crude Model		Adjusted Model	
	OR (95% CI)	*p*	OR (95% CI)	*p*
Food Neophobia				
FNS score	0.997 (0.985–1.008)	0.556	0.989 (0.977–1.002)	0.102
Sociodemographic characteristics				
Age (years)	0.982 (0.961–1.004)	0.103	0.988 (0.965–1.012)	0.330
Sex	0.783 (0.631–0.972)	0.027 *	0.668 (0.532–0.853)	0.001 *
BMI (kg/m^2^)	0.993 (0.964–1.023)	0.651	0.985 (0.951–1.019)	0.383
Education level	0.528 (0.383–0.726)	<0.001 *	0.670 (0.476–0.943)	0.022 *
Awareness and experience				
Awareness of alternative foods	0.264 (0.205–0.340)	<0.001 *	0.365 (0.278–0.480)	<0.001 *
Prior consumption of alternative foods	0.208 (0.163–0.265)	<0.001 *	0.288 (0.220–0.375)	<0.001 *
24 h food-group intake (10 g/day)				
Red meat	1.000 (0.983–1.016)	0.958	1.003 (0.984–1.022)	0.777
Poultry	1.003 (0.992–1.015)	0.604	1.003 (0.990–1.015)	0.671
Potatoes	1.031 (1.012–1.051)	0.001 *	1.027 (1.006–1.048)	0.012 *
Sugary foods	1.053 (1.010–1.098)	0.014 *	1.045 (1.001–1.090)	0.043 *
Processed meat	1.066 (0.986–1.152)	0.108	1.060 (0.989–1.135)	0.098

OR: Odds ratio; CI: Confidence interval; BMI: Body mass index; FNS: Food Neophobia Scale. The adjusted model includes all listed variables entered simultaneously. For dietary variables, ORs represent a 10 g/day increase in intake. Coding of categorical predictors: Sex, female = 0 (reference), male = 1; Education, primary school/high school = 0 (reference), university/postgraduate = 1; Awareness of alternative foods, no = 0 (reference), yes = 1; Prior consumption of alternative foods, no = 0 (reference), yes = 1. * *p* < 0.05.

**Table 5:** Factors associated with unwillingness to try edible insects: crude and adjusted logistic regression models.

Variables	Crude Model		Adjusted Model	
	OR (95% CI)	*p*	OR (95% CI)	*p*
Food Neophobia				
FNS score	1.017 (1.002–1.032)	0.027 *	1.015 (0.998–1.031)	0.077
Sociodemographic characteristics				
Age (years)	1.024 (0.995–1.053)	0.100	1.021 (0.991–1.052)	0.170
Sex	0.669 (0.511–0.875)	0.003 *	0.597 (0.446–0.801)	<0.001 *
BMI (kg/m^2^)	1.003 (0.967–1.041)	0.867	1.008 (0.967–1.051)	0.695
Education level	0.638 (0.435–0.934)	0.021 *	0.782 (0.525–1.165)	0.226
Awareness and experience				
Awareness of alternative foods	0.482 (0.363–0.641)	<0.001 *	0.546 (0.402–0.742)	<0.001 *
Prior consumption of alternative foods	0.564 (0.409–0.780)	<0.001 *	0.683 (0.483–0.967)	0.031 *
24 h food-group intake (10 g/day)				
Red meat	0.998 (0.978–1.019)	0.839	1.002 (0.980–1.024)	0.858
Poultry	0.993 (0.980–1.006)	0.286	0.997 (0.983–1.011)	0.654
Potatoes	0.999 (0.979–1.019)	0.896	0.998 (0.977–1.019)	0.823
Sugary foods	0.963 (0.929–0.998)	0.037 *	0.960 (0.925–0.996)	0.029 *
Processed meat	0.998 (0.942–1.057)	0.942	1.006 (0.952–1.064)	0.822

OR: Odds ratio; CI: Confidence interval; BMI: Body mass index; FNS: Food Neophobia Scale. The adjusted model includes all listed variables entered simultaneously. For dietary variables, ORs represent a 10 g/day increase in intake. Coding of categorical predictors: Sex, female = 0 (reference), male = 1; Education, primary school/high school = 0 (reference), university/postgraduate = 1; Awareness of alternative foods, no = 0 (reference), yes = 1; Prior consumption of alternative foods, no = 0 (reference), yes = 1. * *p* < 0.05.

## Data Availability

The data presented in this study are available on request from the corresponding authors. The data are not publicly available due to privacy and ethical restrictions.

## Supplementary Materials

The following supporting information can be downloaded at: https://www.mdpi.com/article/10.3390/foods15213772/s1, Supplementary Material S1: Study Questionnaire (English Version).
