# Supplementary material for: Beyond Food Neophobia: Factors Associated with Willingness to Consume Cultured Meat and Edible Insects
Source: Foods. 2026 Oct 22;15(21):3772. doi: 10.3390/foods15213772 (PMC13762308; doi:10.3390/foods15213772)
Supplement: Supplementary file 1 [file foods-15-03772-s001.zip › foods-4605235-supplementary.pdf]

Supplementary Material S1

Questionnaire used in the study of cultured meat and edible insect acceptance

The study questionnaire was administered in Turkish.

Terminology note: “Cultured meat” is used in this English translation for the original Turkish term “yapay et”, which interviewers explained as meat produced from animal cells.

A. General information

1. Questionnaire number

____

2. Age

____

3. Sex

____

4. Height (cm)

____

5. Body weight (kg)

____

6. Marital status

1. Single 2. Married

7. Educational level

1. Unable to read or write 2. Able to read and write
3. Primary school 4. Middle school 5. High school
6. University 7. Postgraduate

8. Employment status

1. Employed 2. Not employed

9. Occupation

____

10. Monthly income

1. TRY 0–17,000 2. TRY 17,000–50,000
3. TRY 50,000–100,000 4. TRY 100,000 or more

11. Have you ever lived abroad?

1. Yes 2. No

12. If yes, for how long did you live abroad?

____

B. General health information

1. Do you have any chronic disease(s) diagnosed by a physician?

1. Yes 2. No

2. If yes, please specify the disease(s).

____

3. Do you regularly take any medication? If yes, answer Question B4.

1. Yes 2. No

4. Please write the name of the medication you take.

____

5. Do you smoke?

1. Yes 2. No

6. Do you use dietary supplements? If yes, answer Questions B7 and B8.

1. Yes 2. No

7. How often do you use dietary supplements?

1. Monthly / a few times a year 2. Weekly 3. Daily

8. Please write the name of the dietary supplement you use.

____

C. Dietary habits

1. Which dietary pattern do you follow?

1. Traditional 2. Vegetarian 3. Vegan
4. Flexitarian 5. Pescatarian 6. Other: __________

2. How many meals do you usually eat per day?

Main meals (breakfast, lunch, dinner): _____
Snacks (morning, afternoon, night-time): _____

3. What is your reason for skipping meals?

a. Lack of time b. I do not feel like eating / have no appetite
c. I am dieting d. I forget e. It is not my habit
f. Other: __________

4. On average, how many glasses of water do you drink per day?

________ mL or ________ glasses

5. Are you currently following a diet?

1. Yes 2. No

6. If you are following a diet, what type of diet is it?

____

D. Alternative food questions

1. Have you previously heard of alternative food sources (cultured meat, plant-based milks, edible insects, edible flowers, aquatic plants, microalgae, etc.)?

1. Yes 2. No

2. Where did you hear about alternative food sources?

____

3. Have you previously consumed any alternative food source? If no, go to Question D7.

1. Yes 2. No

4. Please indicate the type(s) of alternative food you have tried.

1. Edible insects 2. Cultured meat 3. Plant-based milk
4. Microalgae 5. Edible flowers
More than one product may be selected.

5. What was your reason for trying alternative food sources?

1. Health benefits 2. Nutrient content 3. Curiosity
4. Vegetarian / vegan diet

5. Environmental concerns / sustainability
6. Other: __________

6. Did you like the taste of the alternative food you tried?

1. Yes 2. No

7. If cultured meat were available for consumption in our country, would you want to consume it?

1. Yes 2. No

8. If no, what is your reason for not wanting to consume cultured meat?

Select one response.
1. Disgust
2. Expected unpleasant taste
3. Religious reasons (e.g., whether the product is halal)
4. I consider it non-nutritious
5. I consider it unhealthy
6. Other: __________

9. Do you think cultured meat could be as safe and healthy as natural meat?

1. Yes 2. No

10. If you had information about the health effects of cultured meat, would you be more inclined to consume these products?

1. Yes 2. No

11. Would you like to try edible insect species?

1. Yes 2. No

12. If no, what is your reason for not wanting to consume edible insects?

Select one response.
1. Disgust
2. Expected unpleasant taste
3. Religious reasons (e.g., whether the product is halal)
4. I consider it non-nutritious
5. I consider it unhealthy
6. Other: __________

13. Do you think edible insects could be as safe and healthy as traditional animal foods?

1. Yes 2. No

14. If you had information about the health effects of edible insects, would you be more inclined to consume these products?

1. Yes 2. No

15. Do you think the nutrient content of alternative foods (cultured meat, edible insects, etc.) is similar to that of conventional products?

1. Yes 2. No

16. Would you prefer alternative foods instead of conventional foods?

1. Yes 2. No

17. Do you think consumption of alternative foods will increase in the future?

1. Yes 2. No

18. Do you think alternative food sources are beneficial for health?

1. Yes 2. No

19. How much extra would you be willing to pay for foods with healthier, personalized nutritional content?

1. 0% 2. 0–50% 3. 50–100% 4. 100% or more

E. Food Neophobia Scale FNS

Tick one box in each row. 1 = Strongly disagree; 2 = Disagree; 3 = Slightly disagree; 4 = Undecided; 5 = Slightly agree; 6 = Agree; 7 = Strongly agree.

Statement	1	2	3	4	5	6	7
1. I am constantly sampling new and different foods.	☐	☐	☐	☐	☐	☐	☐
2. I don’t trust new foods.	☐	☐	☐	☐	☐	☐	☐
3. If I don’t know what is in a food, I won’t try it.	☐	☐	☐	☐	☐	☐	☐
4. I like foods from different countries.	☐	☐	☐	☐	☐	☐	☐
5. Ethnic food looks too weird to eat.	☐	☐	☐	☐	☐	☐	☐
6. At dinner parties, I will try a new food.	☐	☐	☐	☐	☐	☐	☐
7. I am afraid to eat things I have never had before.	☐	☐	☐	☐	☐	☐	☐
8. I am very particular about the foods I will eat.	☐	☐	☐	☐	☐	☐	☐
9. I will eat almost anything.	☐	☐	☐	☐	☐	☐	☐
10. I like to try new ethnic restaurants.	☐	☐	☐	☐	☐	☐	☐

F. 24-h Dietary Recall

Eating occasion	Foods and beverages consumed
Breakfast	
Morning snack	
Lunch	
Afternoon snack	
Dinner	
Night-time snack	
